# New First Line Treatment Options of Clear Cell Renal Cell Cancer Patients with PD-1 or PD-L1 Immune-Checkpoint Inhibitor-Based Combination Therapies

**DOI:** 10.3390/jcm9020565

**Published:** 2020-02-19

**Authors:** Marc-Oliver Grimm, Katharina Leucht, Viktor Grünwald, Susan Foller

**Affiliations:** 1 Department of Urology, University Hospital Jena, 07747 Jena, Germany; katharina.leucht@med.uni-jena.de (K.L.); susan.foller@med.uni-jena.de (S.F.); 2 Interdisciplinary Urology, Western German Tumor Center Essen, Department for Internal Medicine and Urology, University Hospital Essen, 45127 Essen, Germany; viktor.gruenwald@uk-essen.de

**Keywords:** renal cell carcinoma, immune checkpoint inhibitors, axitinib, avelumab, nivolumab, ipilimumab, pembrolizumab

## Abstract

In metastatic renal cell carcinoma (mRCC) the PD-1 immune-checkpoint inhibitor (ICI) Nivolumab became a standard second line treatment option in 2015 based on a significant improvement of overall survival compared to Everolimus. Current pivotal phase 3 studies showed that PD-1 ICI-based combinations were more efficacious than the VEGFR-TKI Sunitinib, a previous standard of care, leading to approval of three new regimens as guideline-recommended first-line treatment. Nivolumab plus Ipilimumab is characterized by a survival advantage, a high rate of complete response and durable remissions in intermediate and poor prognosis patients. Despite frequent immune-mediated side effects, fewer symptoms and a better quality of life were observed compared to Sunitinib. Pembrolizumab or Avelumab plus Axitinib were characterized by an improved progression-free-survival and a high response rate with a low rate of intrinsic resistance. In addition, Pembrolizumab plus Axitinib reached a significant survival benefit. The side effect profile is driven by the chronic toxicity of Axitinib, but there is additional risk of immune-mediated side effects of the PD-1/PD-L1 ICIs. The quality of life data published so far do not suggest any improvement regarding patient-reported outcomes compared to the previous standard Sunitinib. The PD-1/PD-L1 ICIs thus form the backbone of the first-line therapy of mRCC.

## 1. Introduction

The treatment landscape of renal cell carcinoma (RCC) has changed dramatically with the advent of immune-checkpoint inhibition during recent years. In 2019, three PD-1 immune-checkpoint inhibitor (ICI)-based combination regimens were approved as guideline-recommended first-line treatment in metastatic RCC.

The first benchmark leading to the current therapeutical situation was, however, Nivolumab, the first PD-1 ICI to receive FDA and EMA approval after prior therapy in November 2015 and April 2016, respectively. This was based on the results of the CheckMate 025 trial showing:

- Significant improvement of overall survival (26 vs. 19.7 months; hazard ratio (HR) 0.73; *p* = 0.0006)
- Significantly higher objective response rate (26% vs. 5%)
- Better toxicity profile and
- Improved quality of life

These results are compared to a previous second line standard therapy, Everolimus. However, progression-free survival (PFS) was not improved by Nivolumab [1,2]. Subgroup analyses suggested benefits vs. Everolimus independent of age, number of metastatic sites (1 vs. ≥1), location of metastases (bone, liver, lung) as well as number of prior VEGRFR-targeted therapies making Nivolumab a second line standard. The largest improvement is, however, seen in patients with poor prognosis according to the International Metastatic Renal Cell Carcinoma Database Consortium (IMDC; HR for overall survival (OS): 0.48) [3].

The efficacy of PD-1 immune-checkpoint inhibition in patients after prior therapy triggered several clinical trials in the front line setting of metastatic RCC. Three pivotal studies led to approval of PD-1 ICI-based treatment combinations in 2019 and defined new first-line standards [4,5,6].

**Figure 1:**
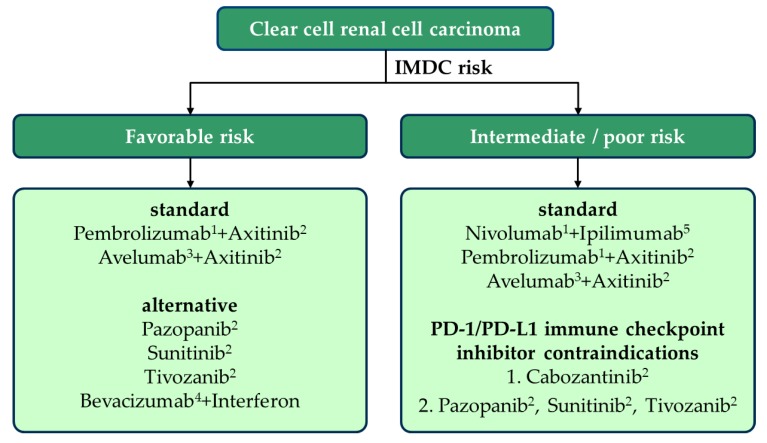
Current therapeutic recommendations for clear cell renal cell cancer; IMDC, International Metastatic Renal Cell Carcinoma Database Consortium. Zero of 6 risk factors (see text): favorable risk, 1–2 of 6 risk factors: intermediate risk, ≥3 of 6 risk factors: poor risk. (1) Anti-PD-1 antibody, (2) VEGFR-TKI, (3) anti-PD-L1 antibody, (4) anti-VEGF antibody, (5) anti-CTLA-4 antibody.

The PD-1 antibody Pembrolizumab and the PD-L1 antibody Avelumab were approved in combination with the VEGFR-TKI Axitinib based upon Keynote 426 [6] and JAVELIN 101 [4], respectively. Additionally, on account of CheckMate 214 [5], for patients with intermediate or poor risk according to the IMDC, the PD-1 antibody Nivolumab was approved in combination with Ipilimumab, a CTLA-4 checkpoint inhibitor (Figure 1).

Due to the new first line regimens, second and further lines of therapy also change. Principally, the 11 formerly available substances are still applicable: old and new VEGFR-TKI and multikinase inhibitors, respectively, as well as the mTOR-inhibitor Everolimus, where appropriate in combination with Lenvatinib [7]. However, there are only few data available that would allow specific recommendations in favor of one of the many therapeutic options.

Hereinafter the current data of the relevant trials will be summarized, focusing on differences between the available combinations. Additionally, possible therapy sequences will be discussed depending on the first-line regimen.

## 2. Risk Stratification of Metastatic Renal Cell Carcinoma

Already during the era of unspecific immunotherapy three risk groups of metastatic RCC, clearly differing regarding OS, had been defined by means of laboratory parameters, performance status and time to necessity of a systemic therapy [8,9]. The IMDC adapted these risk factors and the respective categories for patients receiving VEGFR-TKI [10].

Risk factors according to the IMDC are:

- Karnofsky performance status <80%
- Time between first diagnosis and start of a systemic therapy <1 year
- Low serum hemoglobin
- High corrected serum calcium (corrected serum calcium [mg/dL] = total calcium [mg/dL] + 0.8 (4.0—serum albumin [g/dL]))
- Neutrophilia
- Thrombophilia

Only patients without any risk factors have a relatively good prognosis. If at least three risk factors apply patients belong to a poor prognosis group. Thus, intermediate risk is defined by one to two risk factors.

The IMDC criteria were also used in current pivotal trials comparing new therapeutic approaches against the one first-line standard of care, Sunitinib. Newly tested combination therapies were:

- Nivolumab and Ipilimumab in CheckMate 214 [5]
- Pembrolizumab and Axitinib in Keynote 426 [6]
- Avelumab and Axitinib in JAVELIN 101 [4]

In these three studies patients with advanced or metastatic RCC were eligible. The cohorts differed among others regarding the patient’s IMDC risk profiles: 21%–32% of the patients had favorable, 55%–62% intermediate and 12%–16% high risk tumors. Furthermore, study populations varied regarding the relative contribution of participating countries (Northern America/U.S. vs. Western Europe vs. Rest of the World) and time point of study conduction, both affecting the availability and usage of concomitant and consecutive treatments [4,5,6]. Last but not least, the study endpoints differ as indicated later in the review. Thus, in spite of an apparently equal standard arm, the studies should not be compared in a direct manner.

## 3. Nivolumab Plus Ipilimumab (CheckMate 214)

The combination of Nivolumab 3 mg/kg and Ipilimumab 1 mg/kg is given four-times every three weeks, followed by a maintenance therapy with standard-dose Nivolumab. The co-primary end points of the CheckMate 214 study were objective response rate (ORR), PFS and OS in patients with intermediate and poor prognosis. For Nivolumab and Ipilimumab ORR and OS were increased significantly [5]. Within the three combination trials CheckMate 214 has the longest median follow-up, currently being 32.4 months. The results of the trial are summarized in Table 1. After 30 months, 60% of the patients with intermediate or poor risk were still alive when treated with Nivolumab plus Ipilimumab, as compared to 47% in the treatment arm receiving Sunitinib. As shown for other tumor types treated with this immunotherapy combination PFS-curves form a plateau indicating long-term remission for a considerable number of patients (28% vs. 12% PFS after 30 months of follow-up in Nivolumab plus Ipilimumab vs. Sunitinib). The investigator-assessed ORR was reported to be 42% for Nivolumab plus Ipilimumab with 11% of long-term complete remissions (CRs, 88% of these ongoing) [11].

A limited number of patients with favorable risk (*n* = 250) were included in CheckMate 214. Sunitinib was numerically superior regarding all three co-primary end points (ORR, PFS and OS). Thus, Nivolumab plus Ipilimumab is not approved for patients with favorable risk according to IMDC.

PD-L1 expression in tumor specimens was assessed as a biomarker in CheckMate 214. These data suggest that PD-L1-positive patients have the highest benefit from Nivolumab plus Ipilimumab compared to Sunitinib (PFS HR 0.48; OS HR 0.45). For PD-L1-negative patients’ OS was also increased (HR 0.73) whilst PFS was not [5,11].

## 4. Pembrolizumab Plus Axitinib (Keynote 426)

The co-primary end points of Keynote 426 were OS and PFS in an (IMDC) unselected study population [6]. For Pembrolizumab plus Axitinib, a significant increase of PFS (HR 0.69) and OS (HR 0.59) was achieved, after a median follow-up time of 16.6 months. The ORR was very high (60%, as compared to 38.5% for Sunitinib), complete remission rate was 6.9%. Only very few patients (11.3%) progressed despite treatment with Pembrolizumab plus Axitinib [12,14].

In the initial subgroup analysis all IMDC risk groups seemed to benefit. PD-L1 expression, measured as combined positive score (CPS), appears to be without prognostic impact.

Similar to the other combination trials only few events have occurred in the favorable risk group according to IMDC. Therefore, by now it remains unclear whether this group benefits from the addition of a PD-1 ICI not only in PFS, but also regarding OS (OS HR 0.94 for Pembrolizumab plus Axitinib compared to Sunitinib) [6,12,14].

## 5. Avelumab Plus Axitinib (JAVELIN 101)

Avelumab is a human PD-L1 antibody, which so far has been only approved for metastatic Merkel cell carcinoma and urothelial carcinoma. In the randomized JAVELIN 101 trial, Avelumab plus Axitinib were compared to Sunitinib. The co-primary study endpoints were PFS and OS in PD-L1 positive tumors. Secondary endpoints included OS, PFS, ORR, safety and quality of life in the overall population [4]. The study revealed a significant improvement in PFS in the PD-L1-positive group (HR 0.61) as well as a significant benefit in PFS for the total population (HR 0.69). For OS, data are still immature. So far, no significant benefit for Avelumab plus Axitinib has been demonstrated (HR 0.80; 0.616–1.027 for the total population). The ORR for PD-L1-positive tumors was 55% with 4% of CR. Also for this ICI/VEGFR-TKI combination the rate of progressive disease as best response was low (11%) [13]. Subgroup analyses suggest advantages for combination therapy compared to Sunitinib in all relevant subgroups; the biomarker PD-L1 did not show a clear correlation with clinical outcomes [4,13].

## 6. Side Effects and Quality of Life

The two new first-line combination therapy approaches, immune-immune vs. immune-VEGFR-TKI, differ significantly regarding the side effect spectrum. Axitinib is associated with the well-known chronic VEGFR-TKI toxicity, most commonly diarrhea, hypertension, fatigue, hypothyroidism, hand-foot syndrome (palmar-plantar erythrodysaesthesia syndrome) and gastrointestinal side effects (nausea, loss of appetite, etc.) [15,16]. For ICIs, however, the main focus is on immune-mediated side effects. These mainly affect the skin with rash and pruritus, lung with pneumonitis, intestine with diarrhea and/or immune-mediated colitis, liver with hepatitis (occasionally called transaminitis) and the endocrine system with hyper- or hypothyroidism and, more rarely, hypophysitis or adrenal insufficiency [17]. When a PD-1/PD-L1 ICI is combined with Axitinib, the toxic effects seem to add up numerically. Among others, hepatitis (≥grade 3: ALAT 6.0%–13.3%; ASAT 3.9%–7.0%) and diarrhea (6.7%–9.1% grade 3) may be particularly problematic, not only due to their frequency and severity but also regarding differential diagnosis. For these side effects an immune-mediated etiology is to be diagnosed by excluding other causes, e.g., infectious agents. Overall, adverse events seem to be less frequent with Avelumab plus Axitinib, given the less frequent use of high-dose corticosteroids in the respective pivotal studies (27% with Pembrolizumab plus Axitinib vs. 11.1% with Avelumab plus Axitinib) [4,6].

Regarding patient-reported outcomes (PROs) there are very limited data available for the ICI-VEGFR-TKI combinations. For Pembrolizumab plus Axitinib some PRO data are available from the Assessment Report of the European Medicines Agency (EMA), suggesting disadvantages as compared to Sunitinib in terms of worsening of disease-related symptoms (Functional Assessment of Cancer Therapy Kidney Symptom Index-19 (FKSI-19) disease-related symptoms). In addition, with Pembrolizumab plus Axitinib a worsening in the diarrhea symptom scale of the European Organization for Research and Treatment of Cancer Quality of Life Questionnaire (EORTC-QLQ C30) questionnaire at week 30 compared to baseline was found [14]. Definite conclusions may be drawn after publication of the full quality of life assessments. For Axitinib plus Avelumab, no PROs have been reported so far.

The combination of Nivolumab plus Ipilimumab is associated with a high rate of immune-mediated side effects. These mainly occur during the combination dosings, i.e., during the first 12 weeks. Of the patients 29% received high-dose corticosteroids, 10% for at least 30 days and 19% for at least two weeks. Thus, in contrast to the chronic toxicity caused by a VEGFR-TKI, immune-mediated side effects predominantly occur temporarily. However, side effects concerning the endocrine system are often irreversible and require permanent hormone substitution (thyroid hormones, possibly cortisone) [12]. Patients should be informed about that risk before starting therapy. One in four patients discontinued treatment due to treatment-related adverse events (most frequently transaminitis, diarrhea and pneumonitis), mostly during combination dosings. Nevertheless, 79% of the patients received all four dosings of Nivolumab plus Ipilimumab. However, an analysis of these dropouts suggests that these patients nevertheless benefit from the therapy in terms of response rate and survival [18]. In fact, this and other analyses suggest a relationship between immune-mediated side effects and benefit from the therapy.

For Nivolumab plus Ipilimumab, comprehensive quality of life data (FKSI-19, Functional Assessment of Cancer Therapy-General (FACT-G), and EuroQol five dimensional three level (EQ-5D-3L)) are available from CheckMate 214. These reveal fewer symptoms and a better quality of life as compared to Sunitinib. In fact, quality of life is relatively stable during the period of the combination dosings and subsequently improves as compared to the baseline. As compared to Sunitinib, a sustained improvement in almost all measurements focusing quality of life has been reported [19].

Basically, the treatment of immune-mediated side effects under combination therapy does not differ from that of anti-PD-1/PD-L1 monotherapy. In general, a non-immunological cause must first be identified or excluded for each suspected adverse reaction. For the organ systems that are frequently affected the following procedure can generally be followed according to the common terminology criteria for adverse events (CTCAE):

- Grade 1 therapy can be continued,
- Grade 2: Interrupt therapy, start corticosteroid therapy,
- Grade 4: Permanently discontinue therapy, start corticosteroid therapy.

For grade 3 side effects, the recommendations regarding interrupting or discontinuing therapy are inconsistent; however, for grade 3 pneumonitis or hepatitis, for all approved antibodies a therapy discontinuation is recommended [20].

Most immune-mediated side effects are reversible, provided they are detected early and adequately treated. Exceptions are the endocrine side effects, which may result in a long-lasting or even permanent hormone substitution. This risk should be discussed with patients before starting therapy.

After improvement of a side effect the corticosteroids can be slowly released. If deterioration occurs, an increase in steroid dose or the additional administration of non-steroidal immunosuppressive drugs may be considered. As long as a patient receives immunosuppressive doses of corticosteroids, antibody treatment should be considered. If steroid therapy can be reduced to physiological doses (10 mg methylprednisolone or equivalent) per day, treatment can be continued, given that no discontinuation criteria are met [20,21].

## 7. Second Line Therapy

The use of PD-1/PD-L1 ICIs in the first-line therapy influences the sequential treatment beyond disease progression. Current guideline recommendations regarding sequential therapy are based on data from the VEGFR-TKI era (Level of Evidence IV) [7,22,23]. Moreover, most of these recommendations do not match the approval texts of the corresponding drugs with possible implications regarding reimbursement.

In principle, subsequent to ICI-based combination therapy, all known effective VEGFR-TKIs, if not previously administered (Axitinib), as well as Lenvatinib plus Everolimus may be considered (level of evidence IV) [23]. Many experts favor Cabozantinib as an effective substance in this case, due to its proven advantages over Sunitinib in the first-line as well as due to the (limited) second-line data after PD-1/PD-L1 immune-checkpoint inhibition [24,25,26]. Others consider a modern VEGFR-TKI with a potentially favorable side effect profile such as Tivozanib to be the best choice in the palliative second-line situation [27,28]. Ongoing observational studies will provide further data regarding the known TKIs. Additionally, studies with new substance classes (e.g., HIF-2a inhibitors) will follow.

In patients who receive a VEGFR-TKI targeted first-line therapy (favorable risk patients), Nivolumab or Cabozantinib remain the standard treatment options recommended by current guidelines.

## 8. Non-Clear Cell Renal Cell Carcinoma

The approval of the new combination regimens is, however, not restricted to clear-cell RCC. In general, VEGFR-TKIs appear to be less effective in non-clear cell RCCs. Regarding ICIs there is only little data available. Pembrolizumab monotherapy was analyzed in a phase 2 trial, KEYNOTE-427 (cohort B; *n* = 165). For formerly untreated patients with a papillary (*n* = 118) or unclassified (*n* = 26) RCC histology a considerable ORR (28% and 31%, respectively) was achieved, being higher than expected for a VEGFR-TKI [29]. In contrast, in chromophobe RCC (*n* = 21) the ORR was rather low (9.5%). However, the treatment regimens (immune therapy vs. TKI) were not compared in a direct manner. Currently there are a few randomized trials ongoing in the setting of non-clear cell RCC.

## 9. Conclusions for Clinical Practice

- PD-1/PD-L1 ICIs represent the new backbone of first-line therapy in RCC
- Nivolumab plus Ipilimumab represents a standard first-line therapy for intermediate and high IMDC risk
- Pembrolizumab plus Axitinib or Avelumab plus Axitinib represent standard first-line therapies, regardless of the risk profile
- For patients with a favorable risk profile, a monotherapy with a VEGFR-TKI may be considered as an alternative to the combination therapy
- Patients should be comprehensively advised regarding the advantages and disadvantages of the very different combinations (immune-immune vs. immune-VEGFR-TKI), taking into account the individual situation and therapy objectives.
- Patient information should, among others, include survival benefit, rate of remission, long-term remissions, side effects (chronic side effects of TKIs, risk of immune-mediated adverse events) and quality of life.

## Figures and Tables

**Table 1:** Results of pivotal studies with PD-1/PD-L1 checkpoint inhibitors for first-line treatment of advanced or metastatic renal cell carcinoma.

Clinical Trial	Median Follow-Up	Number of Patients	IMDC Risk	ORR (%)	PFS (Months)	OS (Months)
CheckMate 214 [11] *NCT02231749*	32.4 months	249	favorable	39 vs. 50 (*p* < 0.14)	13.9 vs. 19.9 HR 1.23 (0.90–1.69); *p* = 0.19	NA vs. NA HR 1.22 (0.73–2.04); *p* = 0.44
CheckMate 214 [11] *NCT02231749*	32.4 months	847	Inter-mediate and poor	42 vs. 29 (*p* < 0.0001)	8.2 vs. 8.3 HR 0.77 (0.65–0.90); *p* = 0.0014	NA vs. 26.6 HR 0.66 (0.54–0.80); *p* < 0.0001
Keynote 426 [12] NCT02853331	16.6 months	861	ITT	60.0 vs. 38.5 (*p* < 0.0001)	17.1 vs. 11.1 HR 0.69 (0.57–0.83); *p* = 0.00005	NA vs. NA HR 0.59 (0.45–0.78); *p* = 0.00010
JAVELIN 101 [13] *NCT02684006*	Interim analysis 2	886	ITT	52.5 vs. 27.3	13.3 vs. 8.0 HR 0.69 (0.574–0.825)	NA vs. NA HR 0.80 (0.616–1.027); n.s.
JAVELIN 101 [13] *NCT02684006*	Interim analysis 2	560	PD-L1 positive	55.9 vs. 27.2	13.8 vs. 7.0 HR 0.62 (0.492–0.777)	NA vs. 28.6 HR 0.83 (0.596–1.151); n.s.

HR, hazard ratio; ORR, overall response rate; PFS, progression-free survival; OS, overall survival; NA, not achieved; ITT—intention-to-treat.
